# The full-length mitochondrial genome of *Cobitis nalbanti* (Teleostei: Cypriniformes: Cobitidae)

**DOI:** 10.1080/23802359.2018.1495127

**Published:** 2018-08-13

**Authors:** Hong Keun Park, Keun-Sik Kim, Keun-Yong Kim, In-Chul Bang

**Affiliations:** a Department of Science, Trine University, Angola, IN, USA;; b Ulleungdo-Dokdo Ocean Research Station, Korea Institute of Ocean Science and Technology, Ansan, South Korea;; c AquaGenTech Co., Ltd, Busan, South Korea;; d Department of Life Science and Biotechnology, Soonchunhyang University, Asan, South Korea

**Keywords:** *Cobitis nalbanti*, mitochondrial genome, phylogeny

## Abstract

The entire mitochondrial genome of *Cobitis nalbanti* (Teleostei: Cypriniformes: Cobitidae) was analyzed using the primer walking method. The mitogenome was 16,631 bp in length, comprising 13 protein-coding genes, 2 ribosomal RNA genes, and 22 transfer RNA genes. Its gene order was congruent with those of typical vertebrates. In the phylogenetic tree, *C. nalbanti* was clearly separated from *C. lutheri*, which supported the recent taxonomic revision.

*Cobitis nalbanti* (Teleostei: Cypriniformes: Cobitidae) in South Korea was previously reported as *Cobitis lutheri*, until Vasil’eva et al. (2016) redescribed the latter and erected the former as a new species endemic to Korea due to its morphological and cytogenetic characteristics. In this study, the full-length mitochondrial genome of *C. nalbanti* was analyzed.

A specimen of *C. nalbanti* was collected at Jangpyeong-myeon, Cheongyang-gun in Korea in 2009. The voucher specimen (SCU-0009) was deposited in the aquatic animal collection of the Department of Life Sciences and Biotechnology, Soonchunhyang University (SCU; Asan, South Korea). The genomic DNA was extracted from its fin tissue (Asahida et al. 1996), and the mitogenome was amplified using two independent and overlapping PCR runs, followed by sequencing with a set of 27 primers. The complete sequence was deposited in the GenBank; the accession number MH349461.

Mitogenome sequences of the ‘northern clade’ of the subfamily Cobitinae (Šlechtová et al. 2008) were retrieved from GenBank (http://www.ncbi.nlm.nih.gov/). They were aligned together with the *C. nalbanti* sequence and refined manually to correct obvious misalignments. The nucleotide matrix of 12 protein-coding genes, excluding *nad6*, was created with the first, second, and third bases of each codon. The final matrix comprised 3619 bp for each base. The alignment information is available upon request in FASTA format. Phylogenetic analysis was conducted using RAxML 7.0.4 (Stamatakis 2006) for maximum likelihood (ML) analysis.

The *C. nalbanti* mitogenome was a circular molecule of 16,631 bp in length, consisting of 13 protein-coding genes, 2 ribosomal RNA genes, and 22 transfer RNA genes. The order of these genes was identical to those of corresponding genes that typical vertebrates have.

With this complete mitogenome sequence of *C. nalbanti*, a phylogenetic tree was reconstructed by the ML method, using the nucleotide sequence matrix from 12 concatenated protein-coding genes (Figure 1). In the resulting tree, the cobitid species of the “northern clade” of the subfamily Cobitinae (Šlechtová et al. 2008) were divided into the two lineages. The first lineage included the genera *Misgurnus* and *Paramisgurnus* that did not experience mitochondrial introgression, and the two species of the genus *Koreacobitis*. The second lineage consisted of cobitid species belonging to the genera *Misgurnus* and *Paramisgurnus* that experienced mitochondrial introgression, and the genera *Cobitis*, *Iksookimia*, *Kichulchoia*, and *Niwaellea*, forming a strong monophyletic lineage overall. Among them, *C. nalbanti,* the newly erected species by Vasil’eva et al. (2016) that was formally classified as *C. lutheri* was consistently separated from *C. lutheri*. Therefore, our results supported the taxonomic conclusion of Vasil’eva et al. (2016). Interestingly, our phylogenetic tree supported the monophyletic status of neither *Cobitis*, *Iksookimia*, *Kichulchoia*, nor *Niwaellea*, which is clearly different from the multiple nuclear gene-based tree of Kwan et al. (2018). In the future study, the mitochondrial introgression after hybridization among the species of those genera, as previously observed among some species in the genera *Misgurnus* and *Paramisgurnus* (Šlechtová et al. 2008), should be investigated to clarify their in-depth phylogenetic relationships and evolutionary history.

**Figure 1.**
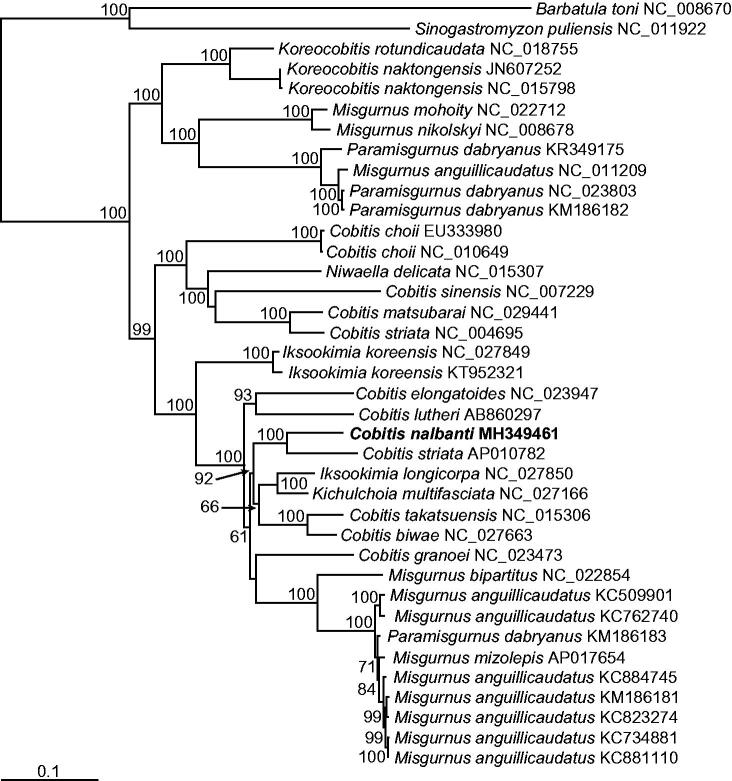
Maximum likelihood (ML) phylogeny based on the full-length mitochondrial genomes from the cobitid species belonging to the subfamily Cobitinae. The matrix included the three codon positions of the 12 protein-coding genes. A bootstrap value above 50% in the ML analysis is indicated at each node. *Cobitis nalbanti* analyzed in this study is shown in bold.
